# Vehicle Cabins as Hotspots of Brominated Flame Retardants: Legacy–Replacement Profiles, Sources, and Human Exposure in a Hot-Climate Environment

**DOI:** 10.3390/jox16030089

**Published:** 2026-05-19

**Authors:** Muhammad Salman Zeb, Mansour A. Alghamdi, Ahmed Summan, Javed Nawab, Muhammad Imtiaz Rashid, Nadeem Ali

**Affiliations:** 1 Department of Environment, Faculty of Environmental Sciences, King Abdulaziz University, Jeddah 21589, Saudi Arabia; mzeb@stu.kau.edu.sa (M.S.Z.); mghamdi2@kau.edu.sa (M.A.A.); asumman@kau.edu.sa (A.S.); 2 Center of Excellence in Environmental Studies, King Abdulaziz University, Jeddah 21589, Saudi Arabia; irmaliks@gmail.com; 3 Department of Environmental and Conservation Sciences, University of Swat Charbagh, Charbagh 19120, Khyber Pakhtunkhwa, Pakistan; jnawab@uswat.edu.pk

**Keywords:** brominated flame retardants, PBDEs, DBDPE, vehicle dust, Saudi Arabia, exposure assessment, human health risk

## Abstract

Brominated flame retardants (BFRs) are widely used in automotive polymers and electronic components, yet vehicles remain an under-characterized and potentially high-exposure microenvironment, particularly in hot climates. This study provides the first comprehensive assessment of BFR occurrence, sources, and exposure risks in vehicle dust from Saudi Arabia, addressing a critical regional data gap. This study systematically investigates the occurrence, compositional patterns, sources, and human exposure risks of polybrominated diphenyl ethers (PBDEs) and selected alternative BFRs in dust from 80 vehicles (domestic cars and taxis; model years 2015–2022) operating in Jeddah, Saudi Arabia. Dust samples were collected using a standardized vacuuming protocol, extracted and cleaned using solvent extraction and silica SPE, and analyzed via GC–NCI–MS. Both legacy PBDE congeners and emerging alternatives (including DBDPE and TBB) were consistently detected, with BDE-209 dominating the overall BFR burden with mean concentrations of 6560 ng/g in domestic vehicles and 5454 ng/g in taxis, with maximum values reaching 220,860 ng/g. Lower-brominated PBDEs occurred at substantially lower concentrations, reflecting the ongoing global transition away from Penta- and Octa-BDE formulations. Taxis exhibited generally higher concentrations than domestic vehicles, likely due to prolonged occupancy, increased usage intensity, and enhanced dust resuspension dynamics. Multivariate analysis (PCA and correlation) revealed two distinct source categories: (i) legacy Penta-BDE-related congeners associated with polyurethane foam and textile materials and (ii) high-brominated PBDEs and DBDPE linked to hard plastics and electronic components. Human exposure assessment demonstrated that dust ingestion is the dominant exposure pathway, while dermal and inhalation routes contribute minimally. Non-carcinogenic hazard indices (HI) were well below unity for all compounds (HI < 1.67 × 10^−6^), and incremental lifetime cancer risks (ILCR) for BDE-209 remained within or near accepted risk thresholds (7.52 × 10^−6^–1.04 × 10^−5^), although occupational exposure among taxi drivers was consistently higher. Overall, the results demonstrate that modern vehicle cabins act as significant microenvironments for chronic BFR exposure, particularly under high-temperature conditions. Despite generally low estimated risks, the combined effects of chemical persistence, bioaccumulation potential, and mixture toxicity—amplified by extreme in-cabin temperatures—highlight vehicles as overlooked yet significant exposure environments. These findings provide the first comprehensive dataset for the Arabian Peninsula and emphasize the need for climate-sensitive exposure assessment, safer material design, and targeted mitigation strategies in vehicle interiors.

## 1. Introduction

Brominated flame retardants (BFRs) constitute a diverse group of synthetic organic chemicals widely incorporated into consumer products to meet flammability standards and reduce fire-related hazards. Their effectiveness has led to extensive use in materials such as textiles, polyurethane foams, electronic components, plastics, and building insulation [1]. To date, approximately 75 different BFRs have been developed, with polybrominated diphenyl ethers (PBDEs) historically being the most dominant class, alongside emerging alternative BFRs such as decabromodiphenyl ethane (DBDPE), 1,2-bis(2,4,6-tribromophenoxy)ethane (BTBPE), and 2-ethylhexyl-2,3,4,5-tetrabromobenzoate (TBB) [2,3,4]. These compounds are added to materials rather than chemically bound to polymers, making them susceptible to release over time through volatilization, abrasion, and diffusion.

Growing scientific evidence indicates that BFRs are ubiquitous contaminants in indoor and outdoor environments, capable of persisting, bio-accumulating, and exerting toxic effects in humans and wildlife. Numerous studies have identified BFRs in human tissues and circulating fluids, revealing exposure through multiple pathways [2,5,6]. PBDEs and some alternative BFRs have been implicated in endocrine disruption, neurodevelopmental toxicity, impaired reproductive function, and altered thyroid hormone signaling [7,8]. Given these concerns, regulatory actions—including restrictions under the EU Directive 2003/11/EC (2003), rulings by the European Court of Justice (2008), and global listing of certain PBDEs under the Stockholm Convention—have driven the phase-out of several PBDE formulations [9,10]. The regulations on PBDE usage have accelerated the adoption of alternative BFRs, whose environmental behavior and toxicological profiles are still not fully understood [4], raising emerging concerns regarding replacement chemicals and their long-term exposure implications.

Indoor environments constitute a major reservoir for BFRs, where these chemicals accumulate in settled dust [11]. Dust ingestion is now recognized as a critical exposure pathway—particularly for children but also relevant for adults—due to the complex composition of dust, which includes fibers, organic debris, cooking emissions, cigarette smoke particulates, and tracked-in soil [11,12,13]. While substantial work has characterized BFRs in household and office dust, comparatively fewer studies have examined their occurrence in vehicles [14,15,16]. This gap is noteworthy because vehicle interiors represent confined microenvironments where drivers and passengers may experience elevated exposures yet remain significantly underrepresented in exposure science and risk assessment frameworks.

Cars incorporate BFR-containing components extensively: polyurethane seat foams, dashboard plastics, electronic modules, interior fabrics, and wiring casings all rely on flame retardants to meet automotive fire safety standards. Despite individuals spending only about 5.5% of their time inside vehicles, concentrations of PBDEs and alternative BFRs in car dust often exceed those in homes [16], suggesting that vehicles may function as disproportionately important microenvironments for human exposure. Elevated temperatures in vehicles, especially in hot climates, can greatly enhance volatilization from treated materials, increasing deposition into dust and potentially facilitating chemical degradation or transformation [17,18]. In hot-climate regions such as Saudi Arabia, in-cabin temperatures can exceed 60 °C, significantly accelerating the emission, migration, and redistribution of BFRs from interior materials. Photolytic reactions may further influence the persistence or breakdown of selected BFRs in dust, as suggested by previous studies investigating indoor and automotive environments [4,16,18].

Understanding BFR behavior in cars is especially relevant for regions such as Saudi Arabia, where vehicles are often exposed to prolonged sunlight and elevated cabin temperatures, accelerating the release and redistribution of BFRs. This high-temperature exposure represents a critical and underexplored factor that may amplify in-cabin contamination and human exposure compared to temperate environments. Under such conditions, drivers may face chronic exposure via ingestion, inhalation, or dermal absorption of BFR-laden dust. Many BFRs exhibit persistence, long biological half-lives, and potential for bioaccumulation, therefore assessing their concentrations in vehicular microenvironments is critical for characterizing exposure risks and informing regulatory and public health strategies.

To address these knowledge gaps, the present study investigates BFRs—including PBDEs and selected alternative BFRs—in dust samples collected from 80 cars manufactured between 2015 and 2022 in Saudi Arabia. This represents one of the most extensive datasets available on BFR contamination in automotive environments, and the first comprehensive examination within the Arabian Peninsula. Importantly, this study focuses on modern vehicle fleets following the global phase-out of major PBDE formulations, providing new insights into the evolving transition from legacy to replacement flame retardants under real-world conditions. The main objective of this study is to comprehensively investigate the occurrence, sources, and human exposure risks of brominated flame retardants (BFRs) in vehicle dust under hot-climate conditions. Specifically, by analyzing the levels, profiles of BFRs, the study aims to (i) assess the extent of contamination in modern vehicle interiors, (ii) characterize spatial and model-related variability in dust concentrations, (iii) evaluate the potential health impacts on drivers who spend prolonged periods inside these vehicles, and (iv) provide region-specific insights considering the uniquely high temperatures of Saudi Arabia, thereby linking chemical occurrence with climate-driven emission processes and human exposure dynamics.

This work contributes crucial baseline information on BFR occurrence in car dust and addresses an urgent need for data in regions where harsh climatic conditions may amplify chemical emissions. The findings provide essential context for evaluating human exposure, informing regulatory frameworks, and guiding strategies to mitigate risks associated with persistent flame retardants in automotive environments. As vehicles serve as both transportation tools and semi-enclosed living spaces, especially in urban areas with long commute times, understanding chemical exposures within this microenvironment is vital for safeguarding public health and supporting evidence-based environmental policies, while also highlighting vehicle cabins as overlooked yet potentially high-impact exposure settings in a warming global climate.

## 2. Materials and Methods

A schematic flowchart summarizing the overall methodology is provided in the Supplementary Materials (Figure S1). The methodological details are described in the following sections.

### 2.1. Chemicals and Solvents for BFR Analysis

Analytical-grade standards for the targeted brominated flame retardants (BFRs) were acquired from AccuStandards (New Haven, CT, USA). These included PBDE congeners 28, 47, 99, 100, 153, 154, 183, and 209, as well as alternative BFRs such as decabromodiphenyl ethane (DBDPE), 2,4,6-tribromophenyl allyl ether (BTBTPE), pentabromobenzyl acrylate (PBBA), hexachlorocyclopentadienyl-dibromocyclooctane (HCCP-DBCO), and hexabromocyclododecane (HBCD). Isotopically labeled internal standards—BDE-77, BDE-128, and ^13^C-BDE-209—were also obtained from AccuStandards and used to ensure reliable quantification and compensate for matrix effects.

Stock solutions of both native and internal standards were prepared in high-purity toluene and iso-octane sourced from Sigma Aldrich (Waltham, MA, USA). Throughout all stages of sample extraction and instrumental analysis, only analytical-grade solvents—including n-hexane, acetone, toluene, ethyl acetate, and iso-octane—were utilized to minimize contamination risks, enhance extraction efficiency, and maintain consistent recoveries, ensuring high analytical reproducibility and data reliability.

### 2.2. Sampling of Floor Dust from Cars (Model Years 2015–2022)

**Figure 1:**
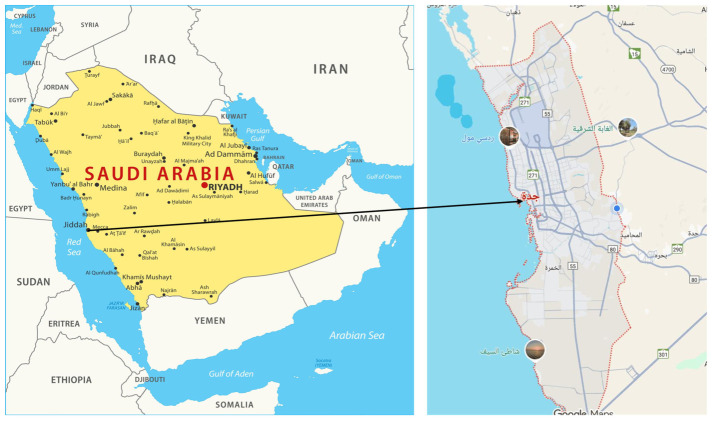
Geographic location of the study area showing a hierarchical map progression from the regional map of Saudi Arabia, indicating the location of Jeddah, and a zoomed view of Jeddah city, where vehicle dust samples were collected.

Dust samples were collected from 80 vehicles operating in Jeddah, Saudi Arabia, with manufacturing years between 2015 and 2022 (Figure 1). The sampled vehicles represented a broad range of makes and models originating from major automotive markets—including China, Japan, Korea, the United States, and the European Union—and included both privately owned vehicles and commercial taxis. To evaluate potential determinants of BFR accumulation, additional information was recorded for each vehicle, such as the average number of hours spent inside the vehicle per day, the frequency of interior cleaning, smoking habits of drivers or passengers, and regular parking conditions (e.g., shaded/covered versus open exposure to sunlight). The sampling questionnaire information is provided in Table S1. The sample size (N = 80) was selected to provide sufficient coverage for assessing BFR occurrence in vehicle dust under real-world conditions rather than based on a priori statistical power calculations. Formal power analysis was not conducted and is acknowledged as a limitation.

A standardized, contamination-controlled sampling procedure was adopted to ensure comparability across vehicles. Dust was collected using a portable vacuum cleaner equipped with a thoroughly washed and pre-cleaned nozzle. A photograph illustrating the sampling setup used for dust collection is provided in the Supplementary Materials (Figure S2). Before sampling each vehicle, the vacuum unit and attachments were thoroughly wiped with high-purity solvent to remove any trace contaminants. Sampling focused on surfaces that commonly accumulate particulate matter, including fabric or leather seats, the trunk area, floor carpets, door panels, and dashboard surfaces, thereby capturing representative dust from multiple in-cabin micro-surfaces. Sampling was conducted under real-world in-vehicle conditions to reflect typical exposure scenarios. Direct measurements of in-cabin thermodynamic parameters (temperature, relative humidity, and solar radiation) were not performed, as the study focused on assessing contaminant levels under representative usage conditions rather than characterizing emission processes. The potential influence of climatic factors was therefore interpreted based on the characteristic environmental conditions of the study area (Jeddah) and supported by relevant literature.

Following collection, the dust samples were sieved through a 200 µm stainless steel mesh to obtain a uniform particle fraction suitable for chemical analysis. The sieved dust was transferred into amber glass vials, tightly sealed, and stored at −20 °C to minimize degradation and potential loss of semi-volatile compounds until further processing. Field blanks were prepared in parallel to assess background contamination. For this purpose, pre-washed anhydrous sodium sulfate was evenly spread on aluminum foil and vacuumed using the same procedure applied to dust sampling. These blanks underwent identical handling, sieving, and storage as vehicle dust samples. The overall sampling approach followed established methodologies previously implemented in studies investigating BFRs in indoor and vehicular environments [15,19], with modifications to ensure consistency across a diverse and modern vehicle fleet.

### 2.3. Sample Preparation and Analysis Procedure

Dust samples were prepared and extracted for BFR analysis using a modified version of the protocol described in [19], with adjustments introduced to improve recovery of the target analytes. Approximately 75 mg of homogenized dust was transferred into pre-cleaned glass tubes, and internal standards (ISs) were added to each sample to account for extraction efficiency and matrix-related effects. A 4:1 (*v*/*v*) mixture of n-hexane and acetone was then introduced, and the samples were left overnight to allow thorough desorption of BFRs from the dust matrix.

Ultrasonication extraction was then applied to the fortified dust samples. Following sonication, the samples were centrifuged, and the resulting supernatants were collected into clean tubes. This extraction step was repeated three times to maximize the recovery of selected BFRs. pooled extracts were carefully concentrated under a gentle nitrogen stream to approximately 100 µL and brought to a final volume of 1 mL using hexane–acetone (1:1, *v*/*v*) mixture. Cleanup of the crude extract was performed using silica BondElut solid-phase extraction (SPE) cartridges. Before use, the cartridges were conditioned, and the BFRs were eluted with 8 mL of n-hexane. The eluates were then evaporated under nitrogen to near dryness and finally reconstituted in 100 µL of iso-octane for GC–MS analysis.

Quantification of BFRs was carried out using a TSQ™ 8000 Evo gas chromatography–mass spectrometry (GC–MS) system (Thermo Scientific, Waltham, MA, USA) operated in negative chemical ionization (NCI) mode. Chromatographic separation was achieved on a TR-5 fused-silica capillary column (30 m × 0.25 mm × 0.25 µm), which provided suitable resolution across the wide range of PBDEs and alternative BFRs analyzed in the study. The injector was set at 230 °C, and the ion source was maintained at 280 °C. Helium of high purity was used as the carrier gas at a constant flow of 1.5 mL/min.

The GC oven program started at 90 °C and then increased at 15 °C/min to 300 °C, where it was held for 1 min. The NCI mode used methane as the reagent gas, which improved molecular ion stability and enhanced sensitivity for highly brominated compounds such as BDE-209 and DBDPE. Data was acquired in selected ion monitoring (SIM) to improve selectivity and quantification precision. Calibration, instrument tuning, and all QA/QC procedures followed previously validated protocols described elsewhere [15,20].

### 2.4. QA/QC (Quality Assurance/Quality Control) Measures

A comprehensive QA/QC framework was applied to ensure analytical robustness and data integrity throughout the study. All laboratory materials were subjected to rigorous decontamination procedures, including detergent washing of glass tubes and high-temperature conditioning, prior to use. Sample handling and preparation were conducted under fume hood without light to minimize contamination and prevent analyte degradation. Analytical performance was continuously evaluated using procedural blanks, field blanks, and matrix-matched spike samples prepared with pre-cleaned sodium sulfate as a dust surrogate. These controls were incorporated at regular intervals within the analytical sequence to track background contamination and method stability. Target compound concentrations were blank corrected where necessary. Isotopically labeled internal standards were added uniformly to all samples and controls to ensure accurate quantification and compensation for potential matrix effects and instrumental variability.

Method accuracy and precision were independently verified using certified reference material (SRM 2585, Gaithersburg, MD, USA, indoor dust) obtained from the National Institute of Standards and Technology. Measured concentrations were in close agreement with established reference values, confirming method trueness. Matrix spike experiments demonstrated consistent extraction efficiency and quantification performance, with recoveries within acceptable analytical ranges. Instrument calibration was routinely checked using multi-level standards, and limits of detection and quantification were determined using signal-to-noise-based criteria. Data quality evaluation included replicate analysis, trend monitoring, and statistical screening for outliers to ensure internal consistency. All deviations from standard operating procedures were recorded and assessed for potential impact on data quality. Collectively, these measures ensured that the reported BFR concentrations in vehicle dust collected from Jeddah are reliable, reproducible, and suitable for high-confidence interpretation in exposure assessment and environmental policy contexts.

### 2.5. Human Risk Assessment Calculations for BFRs

Human exposure to selected BFRs in vehicle dust was evaluated for both non-carcinogenic and carcinogenic risks following established international risk-assessment guidelines [21,22,23]. Chronic daily intake (CDI) through ingestion, inhalation, and dermal contact was calculated using Equations (1)–(3) for non-carcinogenic exposure and Equations (5)–(8) for carcinogenic exposure. All exposure parameters, including intake rates, exposure frequency (EF), exposure duration (ED), body weight (BW), particulate emission factor (PEF), absorption fraction (ABSd), and averaging times (ATnca and ATca), are provided in the Supplementary Information Table S1.

Non-carcinogenic risk was expressed as the hazard quotient (HQ = CDI/RfD) for each pathway, where RfD represents the reference dose recommended by USEPA. The hazard index (HI) was obtained by summing HQ values from all routes (Equation (5)); HI > 1 was considered to indicate potential health concern [11].

CDI ingestion − nca = Cn × (IR × EF × ED/BW × ATnca) × CF(1)

CDI inhalation − nca = Cn × (InhR × EF × ET × ED/PEF × BW× ATnca)(2)

CDI dermal contact − nca = Cn × (SA × SL × ABSd × EF × ED/BW × ATnca) × CF(3)

HQ = CDI − nca/RfD (for each exposure route)(4)

HI = (HQ ingestion + HQ inhalation + HQ dermal contact)(5)

Carcinogenic risk was quantified for BDE 209 as the incremental lifetime cancer risk (ILCR) (Equation (9)) using pathway-specific cancer slope factors (SF) derived from USEPA and WHO databases. An ILCR between 10^−6^ and 10^−4^ was interpreted as an acceptable to tolerable risk range.

CDI ingestion − ca = Cn × (IR × EF/ATca) × CF(6)

CDI inhalation − ca = Cn × (InhR × EF × ET × ED/PEF × 24 × ATca) × 10^3^ (7)

CDI dermal contact − ca = Cn × (ABSd × EF × DFSadj/ATca) × CF(8)

ILCR = CDI ingestion − ca × SF oral + CDI inhalation − ca × SF inhalation + CDI dermal contact − ca × SF dermal(9)

The estimated daily intake (EDI) of BFRs (ng kg^−1^ bw day^−1^) was determined using Equation (10). Two exposure scenarios using mean and 95th percentile concentrations were considered for both regular drivers (2 h day^−1^) representing typical commuter use, and taxi drivers (10 h day^−1^) reflecting occupational long-term vehicle occupancy reported in exposure literature [24]. A standard adult body weight of 70 kg was applied. To describe variability, the mean concentration of BFRs was used for average exposure assessment, while the 95th percentile was used for a worst-case scenario, as recommended by USEPA probabilistic guidance. All equations were verified for dimensional consistency, and parameter units were standardized to ensure that calculated exposure doses (CDI and EDI) were expressed in mg kg^−1^ day^−1^, in accordance with USEPA risk assessment guidelines. A detailed description of parameter units has also been clarified in the Supplementary Information.

Estimated daily intake (ng per kg BW per day) = (Cn × IR/BW) × Ftime (10)

This comprehensive methodology ensured accurate quantification and risk assessment of BFRs in dust samples collected from various car models manufactured between 2015 and 2022, aiding in understanding potential health risks associated with exposure to these compounds.

### 2.6. Statistical Analysis

Statistical analyses were conducted using a combination of spreadsheet-based and specialized data analysis software. Basic descriptive statistics, including detection frequencies, means, medians, maximums, minimums, and standard deviations, were calculated using Microsoft Excel to summarize BFR concentrations in vehicle dust samples. Advanced multivariate analyses were performed using OriginPro 2025 (Version 10.2). Prior to multivariate treatment, concentration data were screened for completeness and consistency, and values below the limit of detection were handled using ½ the value of LOQ. This approach is widely applied in environmental studies when the proportion of non-detects is low and is considered unlikely to introduce significant bias in multivariate analyses. Data were log-transformed where necessary to reduce skewness and approximate normality. Principal component analysis (PCA) was applied to explore patterns in BFR profiles, identify potential source groupings, and assess co-occurrence behavior among target compounds. PCA was performed on normalized datasets using a correlation matrix to minimize the influence of scale differences among variables. The combination of normalization and log-transformation reduces the influence of substituted values and helps preserve the underlying variance structure in PCA. A PMF-like factorization approach based on non-negative matrix factorization (NMF) was also applied to support source apportionment. Component selection was based on eigenvalues greater than unity and visual inspection of scree plots. Loadings and score plots were examined to interpret compound associations and sample clustering. Bivariate correlation analysis was conducted using Pearson correlation coefficients to evaluate linear relationships between individual BFR congeners. Correlation strength and direction were interpreted using established statistical thresholds, and results were used to support source-related inferences suggested by PCA.

## 3. Results and Discussion

### 3.1. Levels and Profile of Brominated Flame Retardants (BFRs) in Vehicle Dust

**Figure 2:**
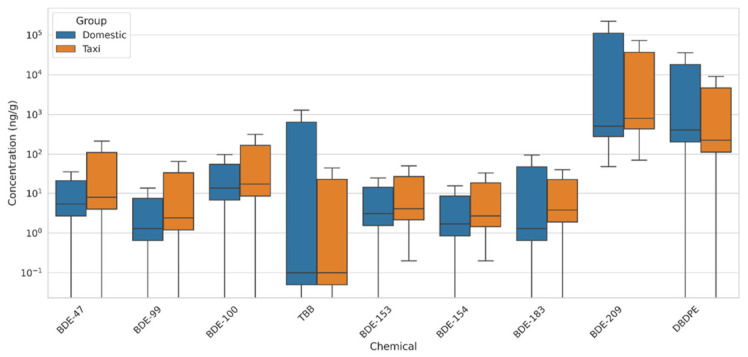
Boxplots comparing concentrations of key BFRs in dust samples collected from domestic cars and taxis. Boxes represent interquartile ranges, with median values indicated and whiskers showing data variability.

The concentrations of BFRs detected in vehicle dust samples from 80 cars in Jeddah, Saudi Arabia, are summarized in Table S2, Figure 2. The results indicate significant variability in the levels of BFRs across different samples, reflecting diverse usage patterns, vehicle types, and exposure conditions.

The concentration patterns of BFRs in vehicle dust from Jeddah revealed pronounced variability between compounds and across vehicle types, highlighting the complexity of flame-retardant use in modern automotive interiors. Consistent with global production and usage trends, BDE-209 emerged as the dominant congener in both domestic and taxi vehicles [14]. Domestic vehicles exhibited a mean concentration of 6560 ± 31,329 ng/g and a median of 508.2 ng/g (range: 49.1–220,860.2 ng/g), while taxis showed similarly elevated levels (mean 5454.5 ± 14,490 ng/g; median 798.9 ng/g). The substantial difference between mean and median values, and the extremely wide concentration ranges, underscore the presence of a small subset of highly contaminated vehicles (Table S1). Such high-end values likely reflect differences in polymer composition of dashboard plastics, seat foams, and electronic casings, as well as the degree of material aging; older or heat-degraded polymers can release BDE-209 more readily [25]. In addition to differences in material composition, release behavior may also depend on material-specific emission mechanisms. In polyurethane foams, repeated compression and friction can promote abrasion-mediated shedding of BFR-containing particles into settled dust [16,26,27]. For hard plastics and polymer blends used in dashboards and interior panels, additive BFRs may migrate from polymer matrices to material surfaces, followed by transfer to dust through volatilization–sorption partitioning or release associated with material wear and micro-cracking [28,29,30]. Moreover, Jeddah’s extreme summer temperatures—often exceeding 60 °C inside parked vehicles—may enhance volatilization and may influence photodegradation of polymer-embedded BFRs, as suggested by previous studies, thereby facilitating their migration into dust and potentially contributing to inter-vehicle variability in contamination levels [18,28,29].

DBDPE, a widely adopted replacement for Deca-BDE, was also detected at appreciable levels, though with somewhat distinct distributional characteristics. Domestic vehicles showed higher DBDPE burdens (mean 1958.7 ± 60,001 ng/g; median 407.1 ng/g) than taxis (mean 853.5 ± 1985.4 ng/g; median 226.2 ng/g). This trend may reflect differential material sourcing between private and commercial vehicle lines; taxis often rely on models with more durable, stain-resistant, and frequently replaced interior components, potentially reducing the long-term accumulation of dust-bound additives. The high standard deviation for domestic vehicles again suggests that a limited number of cars contain interior components heavily loaded with DBDPE-treated polymers—likely variations in specific seat foams, cable insulation, or electronic housings.

Lower-brominated PBDE congeners (BDE-47, BDE-99, BDE-100, BDE-153, BDE-154, and BDE-183) were detected at markedly lower concentrations, reflecting their discontinued global use following regulatory bans on Penta- and Octa-BDE formulations [31,32]. Their presence at ng/g levels likely represents residual contamination from legacy components, cross-contamination during manufacturing, or environmental infiltration rather than ongoing use [31,33]. In taxis, these congeners tended to be slightly higher, possibly due to higher passenger turnover, more abrasive wear of interior surfaces, and more frequent resuspension of dust particles [14,16]. The relatively higher BDE-100 concentrations observed in taxis further support the notion that repeated mechanical stress and cleaning activities may increase the release of legacy BFRs embedded in older or more heavily used seat foams [34]. TBB, a component of alternative flame-retardant formulations used as replacements for Penta-BDE in flexible polyurethane foam, showed a highly skewed distribution in domestic vehicles, with a mean of 74.4 ± 213 ng/g but a median near the detection limit (Table S1). This indicates that TBB is not uniformly present and is likely associated with specific foam or plastic formulations used in certain vehicle models or aftermarket items such as child seats, seat cushions, or interior pads [14,25]. TBB is commonly incorporated into polyurethane foam products as part of replacement mixtures (e.g., Firemaster-type systems), its occurrence in vehicle dust is plausibly linked to the selected interior foam components rather than being a universal automotive additive [35]. The consistently lower concentrations in taxis suggest that commercial fleets tend to use interior materials that either do not rely on TBB-based flame retardants or incorporate alternative systems, and their more frequent cleaning may further reduce dust-bound TBB [36]. The presence of a few vehicles with very high TBB levels supports the interpretation that its use is model-specific, reflecting variation in foam formulations during the transition away from PBDEs toward newer alternative flame-retardant chemistries.

Several emerging BFRs—including HCCP-DBCO, PBBA, and BTBPE—were detected in fewer than 10% of samples. Their low detection frequency is plausible given that many modern flame-retardant formulations are proprietary, and manufacturers selectively incorporate these alternatives into specific polymer systems such as high-performance plastics, wire coatings, or niche electronic parts. Their limited volatility may further restrict migration into dust, and in-car thermal conditions may facilitate rapid photolytic or thermally driven degradation, leaving little measurable residue. Moreover, regional differences in supply chains and the sourcing of automotive interior components influence which BFRs appear in vehicles marketed in Middle Eastern countries. Collectively, the BFR profile in Jeddah’s vehicle dust is characterized by the overwhelming dominance of BDE-209 and DBDPE, the diminishing presence of lower-brominated PBDEs due to regulatory phase-outs, and the sporadic detection of emerging BFRs that reflect an evolving flame-retardant landscape (Figure 2 and Table S1). The pronounced variability observed across vehicles likely arises from a complex interplay of material composition, manufacturing origin, degree of wear and aging, cleaning frequency, and extreme climatic conditions that accelerate chemical release from polymer matrices [33,37]. Given that vehicles constitute enclosed microenvironments where drivers and passengers may spend substantial daily time, the elevated concentrations of high-persistent BFRs highlight the need for continued monitoring of exposure pathways in regions characterized by harsh environmental conditions and diverse vehicle fleets [25]. A Pearson correlation analysis (Figure S3) between vehicle model year and log_10_-transformed BFR concentrations revealed a weak negative trend, suggesting a slight decrease in contaminant levels in newer vehicles. However, the large dispersion of data points indicates substantial variability across individual vehicles, implying that model year alone is not a strong predictor of BFR concentrations. This variability likely reflects differences in material composition, usage patterns, and in-cabin conditions.

### 3.2. Comparison with Global Studies on BFRs in Vehicle Dust

The BFR profile observed in vehicles from Saudi Arabia aligns closely with global trends (Table 1), while also reflecting regional features associated with the country’s relatively young vehicle fleet and extreme climatic conditions. Across international studies, BDE-209 consistently emerges as the dominant congener in vehicle dust, a pattern clearly mirrored in our dataset. Comparable dominance of BDE-209 has been documented in Nigeria [38,39], Egypt [40], the UK [16], the USA [41], Greece [35], Pakistan [14], Kuwait [14,42], Brazil [43], and Czechia [25]. The recurring predominance of BDE-209 reflects its historical and widespread use in ABS, HIPS, and other thermoplastics employed in dashboards, consoles, wiring casings, and electronic housings. Its persistence in modern vehicles—long after regulatory restrictions on PBDEs—underscores the slower global phase-out of Deca-BDE relative to Penta- and Octa-BDE mixtures.

Lower-brominated PBDEs (BDE-47, -99, -100), which originate primarily from Penta-BDE formulations used in polyurethane foams, appeared at comparatively low levels in Saudi vehicles. This pattern is highly consistent with studies from Kuwait and Pakistan [14,42] and with more recent observations from Egypt [40], all of which report diminished presence of Penta-BDE congeners in dust from newer vehicle fleets. By contrast, markedly higher levels have been reported in Nigeria [16,38], the UK [16], and the USA [41], reflecting the prolonged circulation of older vehicles and legacy polyurethane foams treated with Penta-BDE. The relatively modest levels in Saudi Arabia, therefore, indicate that imported vehicles manufactured after the global phase-out of Penta-BDE dominate the market, limiting the contribution of these congeners to indoor car microenvironments.

Mid-brominated congeners such as BDE-153, BDE-154, and BDE-183 showed levels comparable to those reported in Egypt, Kuwait, and Pakistan [14,40,42], and far lower than in regions where older vehicles and legacy materials remain common (e.g., Nigeria and the USA). These trends again reflect differences in fleet age, foam formulations, and regulatory histories that influence the persistence of these congeners in automotive interiors. Emerging BFRs displayed even more pronounced geographical variability. TBB, a component of Firemaster^®^ replacement mixtures used in some polyurethane foams, appeared only sporadically in Saudi dust and at levels far lower than those reported for Greece [35], Kuwait [14], Pakistan [14] and especially Brazil, where extremely high TBB concentrations have been documented [43]. This strongly suggests that TBB-containing formulations are not widely used in the foam systems incorporated into vehicles imported into the Saudi market. DBDPE, the principal global substitute for Deca-BDE, also displayed moderate levels compared with datasets from regions such as Greece or Kuwait [14,35]. This likely reflects regional variation in manufacturing supply chains, with some Asian and Middle Eastern automotive suppliers relying more heavily on traditional Deca-BDE or mixed additive systems than on proprietary DBDPE-based flame-retardant packages.

Taken together, the comparison demonstrates that the BFR profile in Saudi Arabian vehicles most closely resembles those in neighboring Middle Eastern and South Asian markets, where the rapid turnover of imported cars and the predominance of post-2010 models reduce reliance on outdated Penta- and Octa-BDE-treated materials. Meanwhile, the persistent high prevalence of BDE-209—and variable presence of DBDPE—confirms that hard plastics and electronic components remain important contemporary emission sources. The limited occurrence of emerging BFRs such as TBB and the sporadic detection of other proprietary alternatives highlight the strong influence of global supply-chain patterns on chemical use in vehicle manufacturing. Overall, the data underscores how regulatory timing, fleet composition, and regional sourcing of automotive components collectively shape the flame-retardant contamination landscape in vehicle microenvironments.

### 3.3. Multivariate Analysis for Source Identification of BFRs

**Figure 3:**
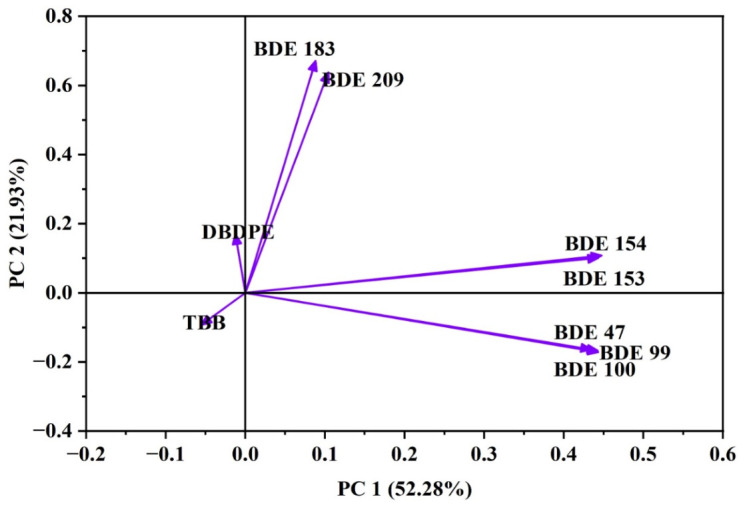
PCA biplot of BFRs in domestic and taxi car dust samples, showing the contribution and distribution of individual compounds along the first two principal components (PC1 and PC2).

Principal Component Analysis (PCA) was conducted to examine co-occurrence patterns among the detected BFR congeners and to identify potential material sources contributing to their presence in vehicle dust. As PCA is a well-established exploratory tool in environmental chemistry, it provides insight into shared emission pathways and helps distinguish BFRs originating from legacy polyurethane foams from those released by modern plastics and electronic components [45]. The first two components had eigenvalues >1 and jointly explained 74.21% of the total variance, with PC1 accounting for 52.28% and PC2 contributing 21.93% (Figure 3). The loading patterns of individual congeners reflect distinct emission sources within the vehicle microenvironment (Figure 3).

PC1 exhibited strong positive loadings for BDE-47, BDE-99, BDE-100, BDE-153, and BDE-154 (loadings > 0.43), indicating a shared emission signature associated with Penta-BDE technical mixtures. These congeners were historically incorporated into polyurethane foam (PUF) materials used in seats, carpet underlays, headliners, and other padded textile-based components [26,27]. Their clustering reflects the continued presence of aged PBDE-treated foam materials within vehicles, consistent with studies showing that mechanical wear, foam degradation, and elevated cabin temperatures increase PBDE migration into vehicle dust [16]. Although BDE-153 and BDE-154 can also originate from Octa-BDE mixtures, their alignment with other Penta-BDE congeners suggests that polyurethane foam remains the dominant emission source for this group in the examined vehicles.

PC2 was characterized by strong positive loadings for BDE-183 (0.67) and BDE-209 (0.64), accompanied by a moderate but positive contribution from DBDPE (0.17). BDE-209 is the principal component of Deca-BDE formulations, historically used in ABS, HIPS, PVC plastics, dashboards, wire insulation, and electronic housings [4,46]. The association of BDE-183 with this component reflects contributions from Octa-BDE mixtures, which were predominantly applied in engineering plastics employed in automotive structural and electronic components [30]. The grouping of BDE-209 with DBDPE aligns with current global trends, as DBDPE has largely replaced Deca-BDE in automotive plastics, particularly in newer vehicle models [46,47]. Their co-loading on PC2 thus indicates that polymeric materials and electronic assemblies represent an important contemporary source of high-brominated BFRs in car dust. TBB and DBDPE plotted close to PCA origin, indicating weak or diffuse associations with either principal component. This pattern likely reflects sporadic use across manufacturers, heterogeneous incorporation in imported vehicle models, and selective application in emerging flame-retardant formulations, such as Firemaster^®^ mixtures used in certain polyurethane foams and molded plastics [10,48]. Their central positioning suggests that their emissions arise from multiple minor sources rather than a dominant material category.

Collectively, the PCA results identify two major BFR source categories in vehicle interiors. The first corresponds to legacy Penta-BDE-treated polyurethane foams, which continue to serve as reservoirs of lower-brominated PBDEs and contribute significantly to dust contamination. The second reflects emissions from Octa-BDE, Deca-BDE, and their replacements (e.g., DBDPE) present in hard plastics and electronic components. These patterns highlight the coexistence of older PBDE-treated upholstery materials and newer polymer-based flame-retardant systems, demonstrating how vehicle age, material composition, and the global transition to alternative BFRs collectively shape exposure patterns inside vehicles. These findings mirror previous observations that vehicles function as hybrid microenvironments—combining both historically used PBDEs and contemporary alternative BFRs—resulting in complex contamination profiles reflective of both past and current manufacturing practices [16,19].

To further validate the source apportionment suggested by PCA, a PMF-like factorization approach (non-negative matrix factorization, NMF) was applied to the dataset. This complementary method enables quantitative identification of contributing source factors and provides additional support for source interpretation (Figure S4). The PMF analysis identified two major source factors. Factor 1 was strongly dominated by BDE-209, indicating emissions from plastic materials and electronic components such as dashboards, wiring insulation, and polymer housings. Factor 2 was characterized by high loadings of DBDPE, with minor contributions from other BFRs, representing replacement flame retardants used in modern vehicle materials. The PMF results are consistent with the PCA findings, which distinguished between legacy PBDE-related sources associated with polyurethane foam and high-brominated compounds linked to polymer-based materials. The agreement between these two independent multivariate approaches strengthens the reliability of the identified source categories and highlights the coexistence of legacy and replacement flame retardants within vehicle interiors.

Although infiltration of outdoor particulate matter may influence dust composition in some driving scenarios, open-window driving is generally uncommon in Jeddah due to routine use of air-conditioned closed-cabin conditions under extreme heat. Therefore, intrusion of road-derived particles through open windows is expected to be limited and unlikely to substantially alter the source patterns observed here, which were dominated by material-associated BFRs linked to interior components. In addition, brand-specific analysis was not conducted due to limitations related to dataset balance and lack of manufacturer-level validation. The inclusion of in-use vehicles introduces confounding factors (e.g., aging, usage intensity, and maintenance), making attribution to specific brands unreliable. Future studies based on controlled sampling of brand-new vehicles may better resolve brand-level differences.

### 3.4. Correlation Analysis of BFRs in Vehicle Dust

**Figure 4:**
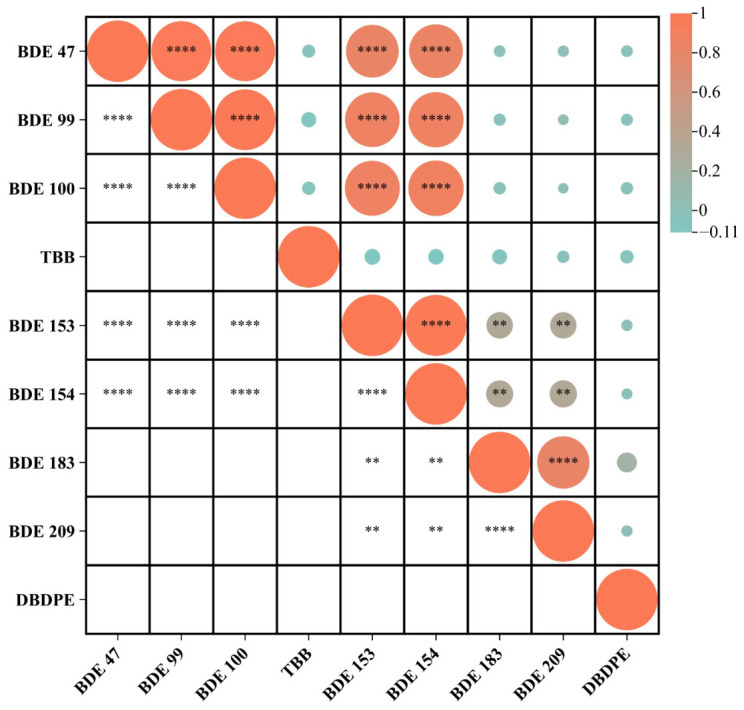
Correlation matrix of BFRs detected in domestic and taxi car dust samples. Circle size and color indicate the strength and direction of correlations, while asterisks denote statistical significance (** *p* < 0.01, and **** *p* < 0.0001).

The correlation matrix (Figure 4) reveals clear co-association patterns among the analyzed BFR congeners, reflecting distinct emission pathways within the vehicle interior. Strong and highly significant positive correlations (*p* < 0.01) were observed among BDE-47, BDE-99, and BDE-100, with correlation coefficients approaching or exceeding 0.80. These congeners also showed strong correlations with BDE-153 and BDE-154, indicating that all five congeners form a coherent cluster. This grouping aligns with their common presence in Penta-BDE technical mixtures, historically used to treat polyurethane foam in vehicle seats, headliners, carpets, and padded textile components [4,26,27]. The strong inter-correlation suggests shared source contributions and similar migration behavior within the confined microenvironment of vehicle cabins, consistent with findings from earlier studies showing that polyurethane foam is a dominant reservoir for these congeners in cars [16].

BDE-183 and BDE-209 displayed significant correlations with each other (*p* < 0.01), reflecting their origin from Octa-BDE and Deca-BDE commercial mixtures, respectively. Their moderate correlations with BDE-153 and BDE-154 further support their partial association with plastic-based automotive materials such as ABS, HIPS, PVC wiring coatings, and electronic housings [30,49]. The correlated behavior of BDE-183 and BDE-209 may also indicate in situ debromination, as BDE-209 is known to degrade into lower-brominated congeners, including BDE-183, under heat and UV exposure—conditions commonly present inside vehicles [14].

In contrast, TBB exhibited weak or non-significant correlations with the PBDE congeners, consistent with its role as a component of alternative flame-retardant formulations, including Firemaster^®^ 550, used in selected polyurethane foams and molded plastics in newer or imported vehicle models [27,50]. DBDPE showed a strong internal correlation (r ≈ 1.0) with itself—as expected from repeated measures in the matrix—but weak or no correlations with PBDE congeners. This pattern reflects its use as a Deca-BDE replacement in modern plastics and electronic casings rather than in polyurethane foam [30,48]. Its weak association with BDE-209 in the correlation analysis (relative to PCA) suggests variable use patterns across vehicle models and manufacturers.

Overall, the correlation analysis supports the presence of two major contamination pathways in vehicle interiors. The first involves legacy Penta-BDE congeners associated with polyurethane foam and textile-based upholstery, and the second involves Octa-/Deca-BDE and DBDPE sourced from hard plastics and electronic components. The weak correlations of emerging flame retardants such as TBB and DBDPE with PBDE congeners underscore their distinct application patterns and the ongoing transition from banned PBDEs toward modern alternatives in automotive manufacturing [4,51].

### 3.5. Human Exposure and Health Risk Assessment

Human exposure assessment showed that ingestion of vehicle dust was the dominant pathway for BFR intake among both domestic and taxi drivers, with dermal absorption contributing moderately and inhalation remaining negligible (Table 2). This exposure hierarchy is consistent with previous studies demonstrating that dust ingestion accounts for the majority of BFR exposure in indoor and vehicular environments, exceeding dermal and inhalation pathways by several orders of magnitude [15,16]. When examining the estimated daily intake values for all congeners, exposures for most compounds remained well below their respective reference doses (RfDs), suggesting limited concern under average exposure conditions (Table 3). In contrast, BDE-209 emerged as the major contributor to aggregate BFR exposure for both driver categories, driven by its elevated concentrations in dust and higher ingestion-based dose. Taxi drivers exhibited substantially higher daily exposures than domestic drivers, reflecting both higher dust burdens in some taxis and longer cumulative time spent inside vehicles. Although total exposure remained below the most conservative RfD benchmarks, the predominance of BDE-209 and the comparatively smaller safety margins in high-end scenarios warrant careful interpretation.

Non-carcinogenic risks were evaluated using HQ and HI across ingestion, dermal, and inhalation pathways (Table 2). In all cases, HQ and HI values were substantially below unity, indicating that chronic non-carcinogenic risks associated with BFR exposure from vehicle dust alone are unlikely to be harmful for adult drivers under current conditions. This aligns with previous assessments showing that indoor-dust-derived PBDE exposure rarely exceeds non-carcinogenic thresholds when evaluated using EPA reference values [15,16,53,54]. Nonetheless, within the overall risk profile, BDE-209 again demonstrated the highest HQs relative to other congeners, consistent with its dominance in the exposure calculations and with reports that BDE-209 frequently drives non-carcinogenic risk in indoor dust [55]. Although current HQ/HI values do not indicate overt health concern, it is important to recognize that many BFRs, including both legacy PBDEs and their replacements, share overlapping toxicological endpoints, particularly regarding thyroid disruption, neurodevelopmental impairment, and endocrine-related modes of action [56]. Traditional HQ/HI frameworks do not capture mixture toxicity, and risk may therefore be underestimated when considering real-world multi-chemical exposures. Although mixture toxicity was considered using the HI approach, the application of toxic equivalency factors (TEFs) is currently limited for PBDEs and alternative BFRs due to the lack of standardized potency factors and differing mechanisms of action. Therefore, the HI approach—widely used for mixtures with similar toxicological endpoints—was considered more appropriate for preliminary cumulative risk assessment in this study. Carcinogenic risk was assessed only for BDE-209 (Table 2), as this is the sole congener with an established oral cancer slope factor [52]. Across all scenarios, incremental lifetime cancer risk (ILCR) values largely fell within or below the commonly accepted benchmark range of 10^−6^–10^−5^ recommended by regulatory agencies. Ingestion was again the predominant contributor, with dermal absorption playing a secondary role and inhalation remaining negligible. Domestic drivers consistently exhibited ILCR values within conservative thresholds, while taxi drivers approached the upper end of the acceptable range under high-exposure (95th percentile) conditions. These results correspond with prior studies showing non-negligible but generally acceptable carcinogenic risk from BDE-209 in dust-rich microenvironments such as vehicles, offices, and public transportation systems [19,57]. However, the cancer slope factor for BDE-209 is associated with considerable uncertainty and variability across regulatory agencies, and the absence of carcinogenicity benchmarks for emerging BFRs—such as TBB and DBDPE—means that the current ILCR approach likely underestimates the total carcinogenic risk [58].

Interpreting these findings within a broader toxicological and public-health context requires careful consideration. Although calculated risk metrics (HI < 1; ILCR ≤ 10^−5^) suggest a low probability of adverse health effects from BFR exposure via vehicle dust alone, the toxicological literature demonstrates clear potential for harmful effects at low doses, particularly following chronic exposure. PBDEs and structurally similar BFRs have been associated with endocrine disruption, altered thyroid hormone homeostasis, immune modulation, and neurodevelopmental deficits in both animal models and human epidemiological studies [59,60,61]. Additionally, vehicle dust is only one of several relevant exposure sources: household dust, dietary intake (especially seafood), indoor air, and occupational environments contribute cumulatively to body burden, meaning the exposures estimated here likely represent a fraction of an individual’s true total intake [62].

The elevated exposures observed by taxi drivers highlight the importance of considering occupational microenvironments in chemical risk assessment. Taxi drivers spend significantly more time inside vehicles, and repeated exposure to elevated dust levels may contribute meaningfully to cumulative BFR intake over the long term. From a risk-management perspective, measures such as regular cleaning of vehicle interiors, reduction in dust accumulation, and selection of automotive materials containing fewer flame-retardant additives could meaningfully reduce exposure, particularly in high-use vehicles. Taken together, the exposure and risk assessment indicates that while current health risks appear within acceptable regulatory limits, the persistence, bioaccumulation potential, and mixture toxicity of BFRs—combined with uncertainties in toxicological benchmarks—underscore the need for continued monitoring and precautionary management, especially for populations with prolonged exposure such as professional drivers.

### 3.6. Limitations

Several limitations should be acknowledged. First, the study was geographically restricted to a single city, which may limit the broader generalizability of the findings to other regions of Saudi Arabia or the wider Middle East. Second, the chemical scope was limited to a selected suite of legacy PBDEs and replacement BFRs; therefore, other emerging flame retardants may also contribute to the overall burden but were not captured here. Third, the study did not include biomonitoring data, which prevents direct linkage between environmental concentrations in vehicle dust and internal human exposure. In addition, although vehicle dust likely reflects in-cabin contamination, contributions from infiltrated outdoor particles could not be fully distinguished from interior-derived dust; however, under the hot climatic conditions of the study area, open-window driving is expected to be limited, so this influence may be relatively small. The exposure assessment also focused on adult scenarios, particularly drivers and taxi drivers, and did not include vulnerable groups such as children or pregnant women, whose exposure profiles may differ because of age-specific behavior and physiological parameters. In addition, thermodynamic parameters such as temperature, relative humidity, and solar radiation were not directly measured; therefore, the influence of heat on BFR emission dynamics and exposure levels was inferred from regional climatic conditions rather than quantified experimentally. In addition, the risk assessment was based on a deterministic approach using mean and high-end exposure scenarios. While this method is widely applied in environmental studies, it does not explicitly capture uncertainty and variability in exposure parameters. Future studies should therefore expand chemical coverage, incorporate biomonitoring, directly measure thermodynamic conditions, include vulnerable populations, and apply probabilistic approaches such as Monte Carlo simulation to provide a more comprehensive evaluation of exposure and risk.

## 4. Conclusions

This study provides the first comprehensive characterization of BFRs in dust from modern vehicles in Saudi Arabia and demonstrates that cars remain an important microenvironment for human exposure to both legacy PBDEs and their contemporary replacements. The BFR profile was dominated by BDE-209 and DBDPE, reflecting their continued use in polymeric automotive components, while lower-brominated PBDEs occurred at comparatively low levels, consistent with the global phase-out of Penta- and Octa-BDE formulations. Taxis generally exhibited higher exposure potential than domestic vehicles due to more intensive use and longer in-cabin occupancy. Multivariate analyses indicated two main sources: polyurethane foam-derived Penta-BDE-related congeners and plastic/electronic components contributing Deca-BDE and DBDPE. Human health risk estimates showed that dust ingestion is the dominant exposure pathway, while inhalation and dermal contact contribute minimally. This is supported by the high concentrations of BDE-209 (mean up to 6560 ng/g; maximum 220,860 ng/g) and low estimated risks (HI < 1; ILCR within 10^−6^–10^−5^), indicating limited immediate health concern. Overall, non-carcinogenic risks were below established thresholds, although BDE-209 contributed most substantially to lifetime cancer risk, particularly among taxi drivers. These findings indicate that current exposure levels do not pose immediate health concerns for adult drivers, but they also highlight the relevance of vehicles as a chronic, daily exposure setting, especially in hot climates where elevated temperatures can accelerate BFR release. Future studies should expand chemical coverage, incorporate biomonitoring, and explore temperature-driven emission dynamics to better understand exposure variability and vulnerable populations. Even in new vehicle fleets, BFR exposure remains ongoing, driven by persistent legacy compounds and modern replacements. Targeted monitoring, improved material choices, and better vehicle hygiene practices could substantially reduce exposure, especially for occupational drivers in regions with extreme heat.

## Figures and Tables

**Table 1:** Comparative concentrations (ng/g) of analyzed BFRs in car dust reported from different countries and years, including taxis and domestic vehicles from Saudi Arabia (current study) and previously published studies worldwide. Note: Values are compiled from published studies; therefore, summary statistics are not consistently reported, and raw data are often unavailable for calculation. Detailed statistics for the current study are provided in Table S2 and Figure 2.

Country	Year	BDE 47	BDE 100	BDE 99	BDE 154	BDE 153	BDE 183	BDE 209	TBB	DBDPE	References
**Saudi** **Arabia**	Taxis—2022	8	17.1	2.4	2.7	4.1	3.8	798.9	0.1	226.2	Current study
	Domestic cars—2022	5.4	13.5	1.3	1.7	3.1	1.3	508.2	0.1	407.1	
**Nigeria**	2013	28	12	49	3.6	9	8.8	780	-	-	[38]
**Nigeria**	2014	68	17	14	19	16	25	122	-	-	[39]
**Egypt**	2013	5.7	4.8	23	3.6	16	5.8	1540	-	-	[40]
**UK**	2009	100	17	130	10	14	6	190,000	-	-	[16]
**USA**	2006	1800	790	2600	120	77	73	3100	-	-	[41]
**Kuwait**	2011	4.3	1.2	7.8	0.8	1.2	2.7	391	-	-	[42]
**Greece**	2017	9.08	1.43	12.2	2.16	3.44	1.39	2830	15.2	848	[35]
**Saudi** **Arabia**	2016	10	2	9	1.5	1.5	2	200	12	280	[15]
**Czechia**	2024	1.97	0.48	3.04	0.46	0.94	3.70	1500	8.50	60.8	[25]
**Brazil**	2017	31.3	0	100	-	0	-	1570	68,200	1360	[43]
**Poland**	2023	0	-	0	-	0	-	600	-	-	[44]
**Pakistan**	2013	1.2	0.30	1.7	0.30	0.90	1.2	625	13	545	[14]
**Kuwait**	2013	5.8	1.5	8.5	1.1	1.5	1.0	665	275	1095	[14]

**Table 2:** Estimated carcinogenic and non-carcinogenic health risk (hazard quotient and hazard index) associated with exposure to BFRs in car dust via relevant exposure pathways. RfDs used for risk assessment were adopted from published literature for PBDEs [11,16,52]. For alternative BFRs, RfD values were derived from available toxicological data and previous exposure assessment studies [14,36], as no regulatory values are currently available.

Non-Carcinogenic								
Chemicals	HQ-Ingestion		HQ-Inhalation		HQ-Dermal		HI	
	Domestic	Taxi	Domestic	Taxi	Domestic	Taxi	Domestic	Taxi
**BDE-47**	3.41 × 10^−7^	1 × 10^−9^	1 × 10^−9^	1.14 × 10^−8^	1.14 × 10^−8^	1.07 × 10^−8^	3.53 × 10^−7^	3.32 × 10^−7^
**BDE-99**	9.39 × 10^−8^	2.76 × 10^−10^	2.76 × 10^−10^	3.15 × 10^−9^	3.15 × 10^−9^	3.46 × 10^−9^	9.74 × 10^−8^	1.07 × 10^−7^
**BDE-100**	5.87 × 10^−7^	1.73 × 10^−9^	1.73 × 10^−9^	1.97 × 10^−8^	1.97 × 10^−8^	2.12 × 10^−8^	6.08 × 10^−7^	6.64 × 10^−7^
**TBB**	1.02 × 10^−8^	3 × 10^−11^	3 × 10^−11^	3.42 × 10^−10^	3.42 × 10^−10^	2.95 × 10^−11^	1.06 × 10^−8^	9.13 × 10^−10^
**BDE 153**	7.05 × 10^−8^	2.11 × 10^−10^	2.11 × 10^−10^	2.4 × 10^−9^	2.4 × 10^−9^	2.24 × 10^−9^	7.31 × 10^−8^	6.88 × 10^−8^
**BDE 154**	9.16 × 10^−8^	2.69 × 10^−10^	2.69 × 10^−10^	3.07 × 10^−9^	3.07 × 10^−9^	2.95 × 10^−9^	9.49 × 10^−8^	9.13 × 10^−8^
**BDE-183**	7.4 × 10^−8^	2.18 × 10^−10^	2.18 × 10^−10^	2.48 × 10^−9^	2.48 × 10^−9^	1.87 × 10^−9^	7.67 × 10^−8^	6.72 × 10^−8^
**BDE 209**	1.17 × 10^−6^	3.43 × 10^−9^	3.43 × 10^−9^	3.9 × 10^−8^	3.9 × 10^−8^	5.41 × 10^−8^	1.21 × 10^−6^	1.67 × 10^−6^
**DBDPE**	1.03 × 10^−8^	3.04 × 10^−11^	3.04 × 10^−11^	3.46 × 10^−10^	3.46 × 10^−10^	1.97 × 10^−10^	1.07 × 10^−8^	6.11 × 10^−9^
**Carcinogenic**								
	ILCR-Ingestion		ILRC-Inhalation		ILCR-Dermal		Total ILCR	
	Domestic	Taxi	Domestic	Taxi	Domestic	Taxi	Domestic	Taxi
**BDE209**	3.89 × 10^−3^	2.22 × 10^−3^	6.33 × 10^−4^	3.61 × 10^−4^	1.24 × 10^−5^	7.12 × 10^−6^	7.52 × 10^−6^	1.04 × 10^−5^

**Table 3:** Estimated daily exposure (ng/kg/day) to BFRs in car dust via dust ingestion. RfDs used for risk assessment were adopted from published literature for PBDEs [11,16,52]. For alternative BFRs, RfD values were derived from available toxicological data and previous exposure assessment studies [14,36], as no regulatory values are currently available.

Target Compounds	RfD (ng/kg/day)	Domestic Drivers		Taxi Drivers	
		Average Daily Exposure	High End Daily Exposure	Average Daily Exposure	High End Daily Exposure
**BDE-47**	100	0.65	3.43	4.76	16.30
**BDE-99**	100	0.15	0.95	1.43	5.25
**BDE-100**	100	1.60	5.95	10.20	32.56
**TBB**	20,000	0.02	20.65	0.06	8.96
**BDE 153**	200	0.37	1.45	2.45	6.78
**BDE 154**	100	0.20	0.95	1.60	4.52
**BDE-183**	200	0.15	1.50	2.25	5.68
**BDE 209**	7000	60.25	1052.50	475.90	5734.05
**DBDPE**	333,333	48.30	347.60	134.70	996.99
**Total BFRs**		**144.90**	**1456.50**	**787.70**	**6253.50**

## Data Availability

The original contributions presented in this study are included in the article/Supplementary Materials. Further inquiries can be directed to the corresponding author.

## Supplementary Materials

The following supporting information can be downloaded at https://www.mdpi.com/article/10.3390/jox16030089/s1: Figure S1. A schematic flowchart summarizing the overall research methodology. Figure S2. Schematic illustration of the vehicle dust sampling protocol, including (a) dust collection from vehicle interior surfaces using a handheld vacuum cleaner, (b) recovery of collected dust from the vacuum collection chamber onto aluminum foil, and (c) sieving of dust samples through a 200 µm stainless steel mesh prior to storage and chemical analysis. Figure S3. Relationship between vehicle model year and log_10_-transformed concentrations of total brominated flame retardants (BFRs) in vehicle dust samples. The red line represents the linear regression (Pearson correlation), indicating a weak negative association between model year and BFR levels. Figure S4. PMF factor profiles of brominated flame retardants (BFRs) in vehicle dust samples (excluding total BFRs). Factor 1 is dominated by BDE-209, representing emissions from plastic and electronic components. Factor 2 is characterized by DBDPE and minor contributions from other compounds, indicating sources associated with replacement flame retardants used in modern vehicle materials. Table S1. Description of parameters used in human health risk assessment equations. Table S2. Descriptive statistics of analyzed BFRs in domestic and taxi car dust samples. All values are provided in ng/g of dust. References [22,63,64,65] are cited in the Supplementary Materials.

## Abbreviations

List of abbreviations and their definitions used throughout the manuscript.

**Abbreviation**	**Definition**
ABS	Acrylonitrile Butadiene Styrene
AT	Averaging Time
ATca	Averaging Time (carcinogenic)
ATnca	Averaging Time (non-carcinogenic)
BDE	Brominated Diphenyl Ether
BFRs	Brominated Flame Retardants
BTBPE	1,2-bis(2,4,6-tribromophenoxy)ethane
BW	Body Weight
CDI	Chronic Daily Intake
CF	Conversion Factor
DBDPE	Decabromodiphenyl Ethane
ED	Exposure Duration
EDI	Estimated Daily Intake
EF	Exposure Frequency
ET	Exposure Time
GC–NCI–MS	Gas Chromatography–Negative Chemical Ionization–Mass Spectrometry
HBCD	Hexabromocyclododecane
HCCP-DBCO	Hexachlorocyclopentadienyl-dibromocyclooctane
HI	Hazard Index
HQ	Hazard Quotient
ILCR	Incremental Lifetime Cancer Risk
InhR	Inhalation Rate
IR	Ingestion Rate
IS	Internal Standard
LOQ	Limit of Quantification
PBBA	Pentabromobenzyl Acrylate
PBDEs	Polybrominated Diphenyl Ethers
PCA	Principal Component Analysis
PEF	Particulate Emission Factor
PUF	Polyurethane Foam
QA/QC	Quality Assurance/Quality Control
RfD	Reference Dose
SA	Skin Surface Area
SF	Slope Factor
SIM	Selected Ion Monitoring
SPE	Solid Phase Extraction
TBB	2-ethylhexyl-2,3,4,5-tetrabromobenzoate
USEPA	United States Environmental Protection Agency
