# Supplementary material for: Vehicle Cabins as Hotspots of Brominated Flame Retardants: Legacy–Replacement Profiles, Sources, and Human Exposure in a Hot-Climate Environment
Source: J Xenobiot. 2026 May 19;16(3):89. doi: 10.3390/jox16030089 (PMC13214825; doi:10.3390/jox16030089)
Supplement: Supplementary file 1 [file jox-16-00089-s001.zip › jox-4284367-supplementary.pdf]

Journal of Xenobiotics

Supplementary Materials: Vehicle Cabins as Hotspots of Brominated Flame Retardants: Legacy–Replacement Profiles, Sources, and Human Exposure in a Hot-Climate Environment

Muhammad Salman Zeb, Mansour Ahmed Alghamdi, Ahmed Summan, Javed Nawab, Muhammad Imtiaz Rashid, Nadeem Ali

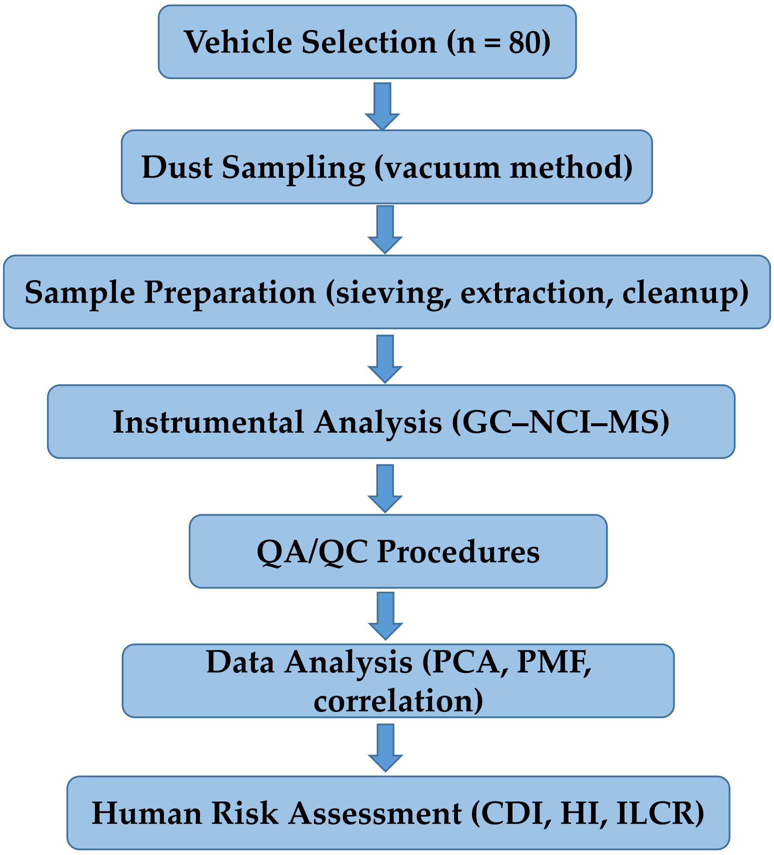

Figure S1. A schematic flowchart summarizing the overall research methodology.

 https://doi.org/10.3390/jox16030089

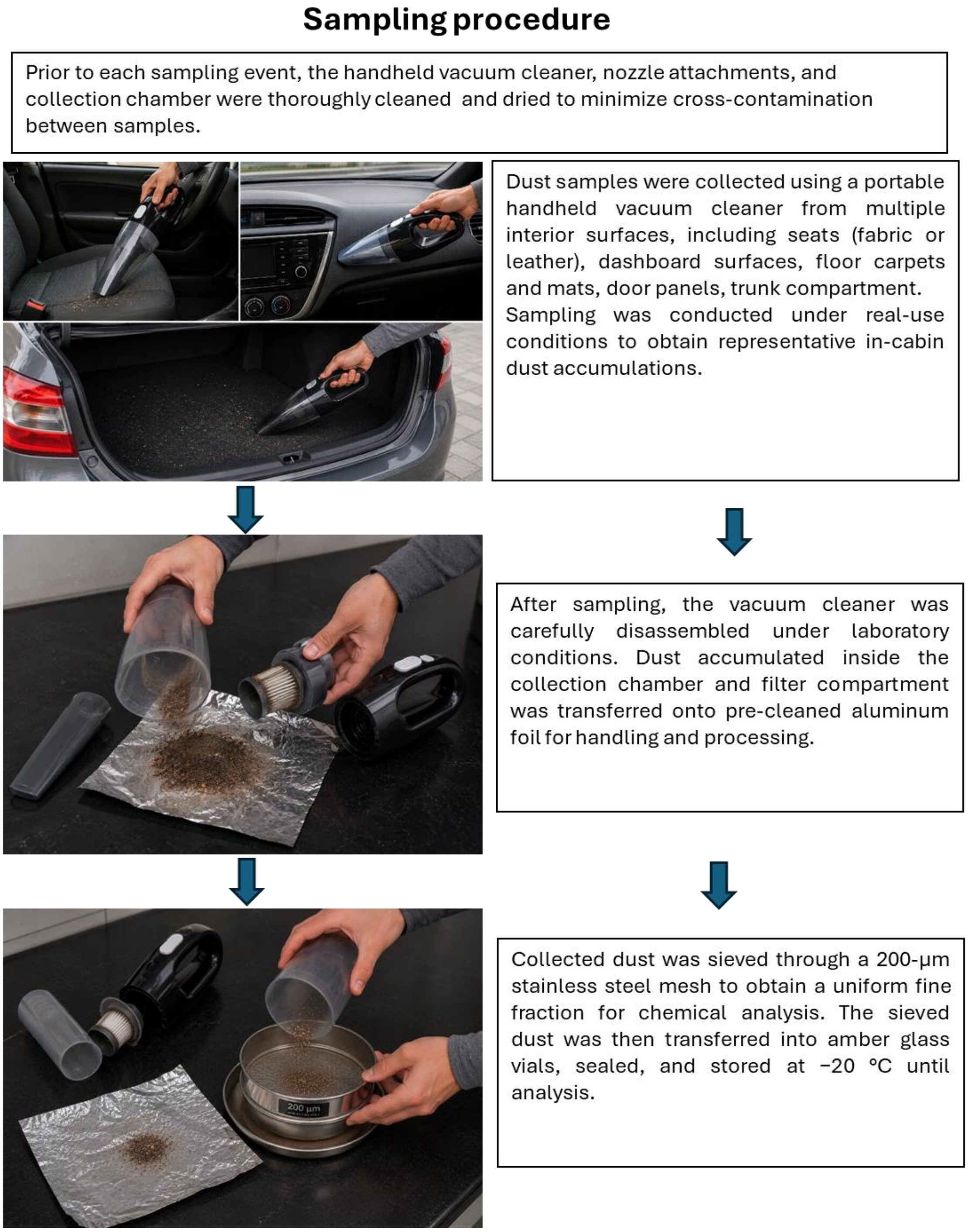

Figure S2. Schematic illustration of the vehicle dust sampling protocol, including (a) dust collection from vehicle interior surfaces using a handheld vacuum cleaner, (b) recovery of collected dust from the vacuum collection chamber onto aluminum foil, and (c) sieving of dust samples through a 200-μm stainless steel mesh prior to storage and chemical analysis.

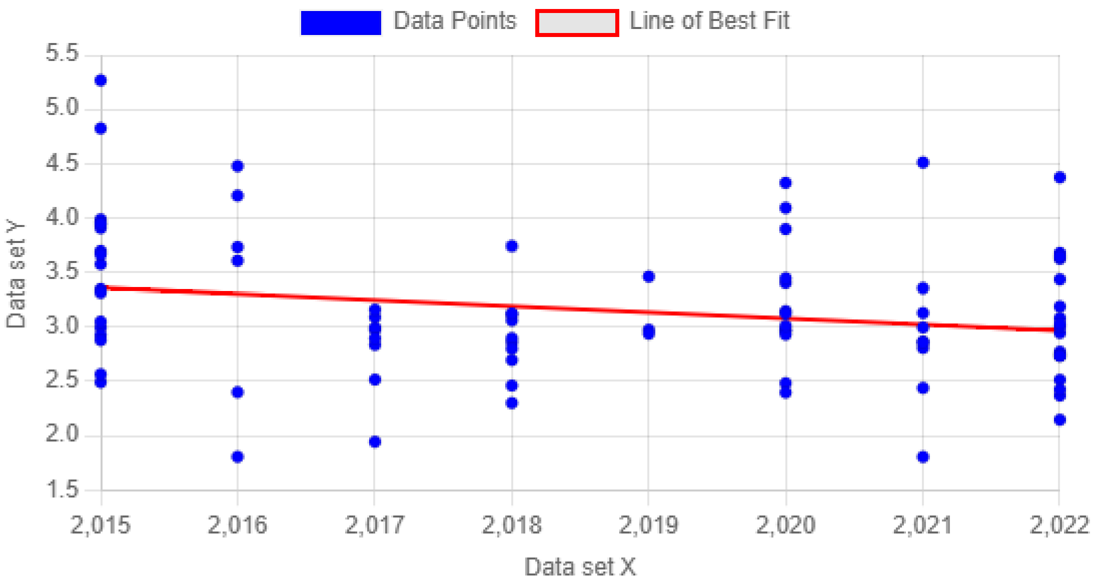

Figure S3. Relationship between vehicle model year and log_{10}-transformed concentrations of total brominated flame retardants (BFRs) in vehicle dust samples. The red line represents the linear regression (Pearson correlation), indicating a weak negative association between model year and BFR levels.

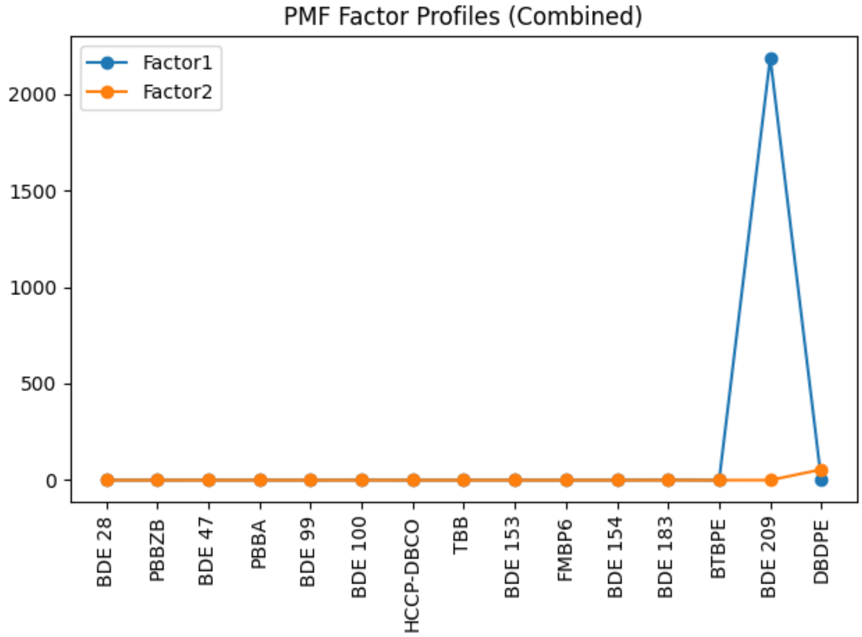

Figure S4. PMF factor profiles of brominated flame retardants (BFRs) in vehicle dust samples (excluding total BFRs). Factor 1 is dominated by BDE-209, representing emissions from plastic and electronic components. Factor 2 is characterized by DBDPE and minor contributions from other compounds, indicating sources associated with replacement flame retardants used in modern vehicle materials.

Table S1. Description of parameters used in human health risk assessment equations.

Parameters	Domestic	Taxi	Reference
Ingestion rate (IR) (mg d^{-1})	100	100	[63]
Inhalation rate (InhR) (m^3 d^{-1})	20	20	[63]
Exposure frequency (EF) (d $year^{-1}$)	350	350	[21]
Duration of exposure (ED) (years)	30	30	[64]
Exposed skin area (SA) (cm^3)	6700	6700	[64]
Dust to skin adherence factor (SL) (mg cm^{-2})	0.5	0.5	[64]
Dermal absorption factor (ABSd)	0.001	0.001	[21]
Particle emission factor (PEF) (m^3 kg^{-1})	1.36×10^9	1.36×10^9	[21]
Body weight (BW) (kg)	70	70	[65]
Lifetime (LT) (years)	70	70	[21]
Conversion factor (CF)	1×10^{-6}	1×10^{-6}	[21]
Dust dermal contact factor, age-adjusted (DFSadj)	362.4	362.4	[21]
Dust ingestion rate, age-adjusted (IR) (mg×year kg^{-1} d^{-1})	113	113	[21]
Exposure time (ET) (h d^{-1})	20	20	[64]
Average non-carcinogenic exposure time (ATnca)	ED × 365	ED × 365	[21]
Average carcinogenic exposure time (ATca)	LT × 365	LT × 365	[21]

Table S2. Descriptive statistics of analyzed BFRs in domestic and taxi car dust samples. All values are provided in ng/g of dust.

Chemicals	Domestic		Taxi	
	Mean ± StDev	Median (Min-Max)	Mean ± StDev	Median (Min Max)
BDE 47	10.2 ± 11.3	5.4 (BDL -36.2)	20.0 ± 38.7	8.0(BDL-214.5)
BDE 99	2.8 ± 3.3	1.3 (BDL -13.6)	6.4 ± 12.5	2.4(BDL-66)
BDE 100	21.2 ± 20	13.5 (BDL -98)	38.0 ± 60.3	17.1(5.1-317.7)
TBB	74.4 ± 213	0.1 (BDL – 1282.7)	4.5 ± 10	0.1(BDL-45.3)
BDE 153	5.3 ± 5.7	3.1 (BDL – 25.4)	7.2 ± 9.6	4.1(0.2-51)
BDE 154	3.1 ± 3.6	1.7 (BDL – 15.4)	4.7 ± 6.4	2.7(0.2-34)
BDE 183	6.6 ± 16	1.3 (BDL – 95.7)	5.3 ± 7	3.8(BDL-40.8)
BDE 209	6560 ± 31329	508.2 (49.1 – 220860.2)	5454.5 ± 14490	798.9(71-72607.8)
DBDPE	1958.7 ± 60001	407.1 (BDL – 35800.5)	853.5 ± 1985.4	226.2(BDL-9106.1)

References

21 USEPA, 2011. Exposure factors handbook. Office of research and Development, Washington, DC, 20460, pp.2-6.
63 Ali, N., 2019. Polycyclic aromatic hydrocarbons (PAHs) in indoor air and dust samples of different Saudi microenvironments; health and carcinogenic risk assessment for the general population. *Science of the Total Environment*, *696*, p.133995.
64 Australian Exposure Factor Guidance Document, Guidelines for Assessing Human Health Risks from Environmental Hazards. Department of Health. 2011. Available online: https://www.health.gov.au/internet/main/publishing.nsf/content/A12B57E41EC9F326CA257BF0001F9E7D/$File/Aust-Exposure-Factor-Guide.pdf (accessed on 19 January 2026).
65 Albar, H.M.S.A., Ali, N., Eqani, S.A.M.A.S., Alhakamy, N.A., Nazar, E., Rashid, M.I., Shahzad, K. and Ismail, I.M.I., 2020. Trace metals in different socioeconomic indoor residential settings, implications for human health via dust exposure. *Ecotoxicology and environmental safety*, *189*, p.109927.

Disclaimer/Publisher's Note: The statements, opinions and data contained in all publications are solely those of the individual author(s) and contributor(s) and not of MDPI and/or the editor(s). MDPI and/or the editor(s) disclaim responsibility for any injury to people or property resulting from any ideas, methods, instructions or products referred to in the content.
